# The PIRAGUA_indicators dataset: Streamflow indices of the Pyrenees rivers (1950–2019)

**DOI:** 10.1016/j.dib.2026.113159

**Published:** 2026-08-17

**Authors:** Leticia Palazón, Ane Zabaleta, Iñaki Antigüedad, Luis Javier Lambán, Jorge Jódar, Vivien Hakoun, Pierre Le Cointe, Yvan Caballero, Santiago Beguería

**Affiliations:** a Estación Experimental de Aula Dei (EEAD), Consejo Superior de Investigaciones Científicas (CSIC), Avda. Montañana 1005, 50059, Zaragoza, Spain; b Hezkuntza, Filosofia eta Antropologia Fakultatea, HEFA II, Euskal Herriko Unibertsitatea (UPV/EHU), Plaza Oñati 3, 20018 Donostia-San Sebastián, Spain; c Zientzia eta Teknologia Fakultatea, Euskal Herriko Unibertsitatea (UPV/EHU), Barrio Sarriena s/n, 48940, Leioa, Spain; d Instituto Geológico y Minero de España (IGME), Consejo Superior de Investigaciones Científicas (CSIC), Avda. Montañana 1005, 50059, Zaragoza, Spain; e INRAE, UMR 1114 EMMAH INRAE-Avignon University, Domaine Saint-Paul, Site Agroparc, 228 Route de l'Aérodrome, CS 40509, 84914 Avignon Cedex 9, France; f BRGM – French Geological Survey, Parc Technologique du Canal, 3 Rue Marie Curie, BP 49, 31527, Ramonville-Saint-Agne, France; g BRGM – French Geological Survey, 1039 Rue de Pinville, 34000, Montpellier, France; h G-EAU, Univ Montpellier, AgroParisTech, BRGM, CIRAD, INRAE, Institut Agro, IRD, 361 Rue Jean-François Breton, 34196, Montpellier, France

**Keywords:** Daily discharge, Groundwater levels, Hydrological variability, Trend analysis, Long-term analysis, Mountain hydrology, Transboundary monitoring Spain, France, Andorra

## Abstract

This paper presents a database of descriptive statistics and trend analyses performed on long-term records of streamflow (93 stations) and groundwater levels (29 monitoring wells) across the Pyrenean region (France, Spain and Andorra). Whereas streamflow time series generally cover several decades, groundwater level records are shorter and often discontinuous, yet they provide valuable insights into aquifer dynamics. The dataset includes 19 streamflow and groundwater indicators based on daily data, representative of mean, high, and low flows, allowing a complete characterisation of streamflow regimes of the Pyrenean rivers. Additionally, trend magnitudes and significance levels, derived using standardized non-parametric tests, are provided for each of the indicators. The analyses were conducted over multiple time windows of different lengths, all ending in 2019, and applied at both annual (hydrological year: October–September) and monthly scales. The set of indicators provides a consistent framework for assessing spatial and temporal patterns of hydrological change in mountain catchments.

Specifications Table

**Table:** 

Subject	Earth & Environmental Sciences
Specific subject area	Hydrology and Water Quality; Water Science and Technology; Daily streamflow; Aquifer level; Streamflow and groundwater indicators; Trend analysis.
Type of data	Plain text files, PDF files and GIS shapefiles.
Data collection	The dataset was generated from quality-controlled daily streamflow records and groundwater-level observations provided by official monitoring agencies in Spain, France and Andorra. Streamflow indicators and groundwater statistics were derived from the original time series using standardized statistical procedures. Monitoring stations were selected according to data quality, availability and temporal coverage to ensure robust long-term analyses. The processed data were subsequently compiled into tabular, spatial and graphical products for dissemination.
Data source location	• Region: Pyrenees. • Countries: Spain, Andorra and France. • Coordinates (latitude/longitude): area limited by 40.615/-4.536 and 45.626/4.505, according to the coordinate system WGS84 (EPSG: 4326).
Data accessibility	Repository name: Digital.CSIC Data identification number: 10.20350/digitalCSIC/14658 Direct URL to data: http://hdl.handle.net/10261/270751
Related research article	A. Zabaleta, L. Palazón, I. Antigüedad, S, Beguería, 2026. Changing rivers: Hydrological shifts in the Pyrenees revealed by daily streamflow indicators (1950–2019). J. Hydrol.: Reg. Stud., 65, 103374. https://doi.org/10.1016/j.ejrh.2026.103374 [1]

## 1 Value of the Data

• The dataset provides harmonized annual and monthly hydrological indicators derived from long-term daily streamflow records collected at 93 reference gauging stations across the Pyrenees, together with groundwater-level indicators from 29 monitoring wells.
• It includes trend magnitudes and significance statistics calculated for multiple temporal windows (1950–2019 to 2000–2019), enabling consistent temporal comparisons among stations and indicators.
• The dataset combines tabular data, GIS layers, maps and station reports, facilitating reproducibility, data visualization, and spatial analyses.
• The standardized indicators can be directly reused for regional hydrological assessments and comparative studies with other mountain regions.

## 2 Background

Long-term hydrological observations are essential for documenting changes in water resources and for evaluating the effects of climatic and environmental change on mountain catchments. The Pyrenees constitute a major water source for southwestern Europe, supplying water to the Ebro, Garonne, Adour and several Atlantic and Mediterranean coastal basins. Previous studies have reported changes in streamflow regimes, snow dynamics, drought occurrence and water availability in different sectors of the mountain range [[2], [3], [4], [5], [6]].

Although numerous hydrological datasets are available from national and regional monitoring networks, data coverage, formats and analytical approaches differ among institutions and countries. As a result, regional-scale assessments covering the entire Pyrenean range remain difficult to perform. In addition, most previous analyses have relied on monthly or annual data, whereas daily records allow the derivation of indicators describing different components of the flow regime, including low-flow, median-flow and high-flow conditions.

The *PIRAGUA_indicators* dataset was developed to provide a harmonized compilation of hydrological indicators and trend statistics derived from long-term streamflow and groundwater records collected across the Pyrenees. The dataset contains annual and monthly indicators, trend estimates and significance statistics calculated using standardized procedures, together with GIS layers and graphical products that facilitate spatial analyses and data interpretation. It complements the hydrological assessment presented by Zabaleta et al. [1], by providing open access to the derived indicators, metadata and trend-analysis outputs associated with that work.

## 3 Data Description

The PIRAGUA_indicators dataset contains hydrological indicators and trend statistics derived from long-term streamflow and groundwater records collected across the Pyrenees by different agencies. Streamflow information was obtained from 93 reference gauging stations distributed throughout Spain, France and Andorra, whereas groundwater information was derived from 29 monitoring wells with sufficient temporal coverage to allow for trend analysis. The database includes annual and monthly indicators describing flow magnitude, variability, low-flow conditions and high-flow conditions, together with estimates of trend magnitude and statistical significance.

The dataset is distributed through the DIGITAL.CSIC repository and is organized into eight compressed files (.zip) containing plain text tabular data (.txt), GIS layers (.shp, requiring Geographic Information System, GIS, software) and graphical outputs (.pdf). Tabular files provide hydrological indicators and trend statistics for different temporal windows ending in 2019, while GIS layers contain the spatial location of monitoring sites, catchment boundaries and aquifer units. Additional PDF products summarize the spatial distribution of indicators and trends and provide individual reports for monitoring stations. All files are accompanied by metadata describing variables, units and file structure. The dataset is organized into the following compressed folders:

• **groundwater_maps_trends.zip** – PDF maps summarizing groundwater trend analysis.
• **groundwater_means.txt** – Average values of groundwater indices at selected monitoring stations for 1995–2019 temporal window.
• **groundwater_trends.txt** – Trend magnitudes and significance levels of groundwater indices for 1995–2019 temporal window.
• **streamflow_maps_trends.zip** – PDF maps summarizing streamflow trend analysis.
• **streamflow_means.txt** – Average values of daily streamflow indicators at selected gauging stations over multiple analysis windows (1950–2019, 1960–2019, 1970–2019, 1980–2019, 1990–2019, 2000–2019).
• **streamflow_trends.txt** – Trend magnitudes and significance levels of daily streamflow indicators for the same analysis periods.
• **streamflow_station_reports.zip** – Individual PDF reports for each streamflow gauging station, organized by temporal window.
• **gis.zip** – Shapefiles containing the geographic location (EPSG: 25830) of streamflow (*streamflow_stations.shp*) and groundwater monitoring stations (*groundwater_stations.shp*), streamflow station contributing catchments (*gauge_catchment.shp*), and main aquifer boundaries (*aquifers.shp, aquifers_types.shp and aquifers_confinement.shp*).

All tabular files are provided as tab-delimited text files and can be imported directly into common statistical and GIS software environments. Variable names, units and identifiers are consistent across files, allowing direct integration of the different dataset components. Variables in the data files:

• *time*: temporal support of the data (Annual, January, ..., December).
• *station*: gauging station id, corresponds to the id in the shapefile.
• *index*: streamflow index (see list of indices below).
• *units*: units, either absolute (m^3^ s^−1^) or relative (mm).
• *epoch*: temporal window over which the index has been calculated. One of (1950-2019, 1960-2019, 1970-2019, 1980-2019, 1990-2019, 2000-2019).
• *value*: index value. Only in file `streamflow_means.txt`.
• *trend*: trend magnitude, according to the Theil-Sen's method (m^3^ s^−1^ or mm per decade). Only in file `streamflow_trends.txt`.
• *trend_r*: trend magnitude, according to the Theil-Sen's method (m^3^ s^−1^ / m^3^ s^−1^ or mm / mm per decade). Only in file `streamflow_trends.txt`.
• *sig*: significance of the trend, according to (pre-whitenned) Kendall test. Only in file `streamflow_trends.txt`.

Streamflow indicators:

• *mean*: mean daily streamflow (m^3^ s^−1^ or mm).
• *sd*: standard deviation of daily streamflow (m^3^ s^−1^ or mm).
• *max*: maximum daily streamflow (m^3^ s^−1^ or mm).
• *min*: minimum daily streamflow (m^3^ s^−1^ or mm).
• *q10*: 10th percentile of daily streamflow (m^3^ s^−1^ or mm).
• *q25*: 25th percentile of daily streamflow (m^3^ s^−1^ or mm).
• *q50*: 50th percentile of daily streamflow (median) (m^3^ s^−1^ or mm).
• *q75*: 75th percentile of daily streamflow (m^3^ s^−1^ or mm).
• *q90*: 90th percentile of daily streamflow (m^3^ s^−1^ or mm).
• *iqr*: inter-quartile range (*q75*-*q25*) (m^3^ s^−1^ or mm).
• *uqr*: upper-quartile range (*q75*-*q50*) (m^3^ s^−1^ or mm).
• *lqr*: lower-quartile range (*q50*-*q25*) (m^3^ s^−1^ or mm).
• *qsk*: quantile skewness (*uqr*-*lqr*); > 0 for right skewness [-1, 1].
• *idr*: inter-decile range (*q90*-*q10*) (m^3^ s^−1^ or mm).
• *dsk*: decile skewness ((*q90* - *q50*) - (*q50* - *q10*)) / (*q90* - *q10*); > 0 for right skewness; range [-1, 1].
• *dku*: decile kurtosis (*q90*-*q10*) / (*q75*-*q25*).
• *vcn3*: baseflow index, daily minimum streamflow averaged over 3 consecutive days (m^3^ s^−1^ or mm).
• *vcn7*: baseflow index, daily minimum streamflow averaged over 7 consecutive days (m^3^ s^−1^ or mm).
• *rl20*: streamflow corresponding to a 20-years return period (m^3^ s^−1^ or mm).

## 4 Experimental Design, Materials and Methods

Raw data (daily streamflow and monthly or irregular groundwater level time series) were collected from the official observation networks of the different administrations with jurisdiction over water resources in the Pyrenean territory: *Red Oficial de Estaciones de Aforo* (ROEA; https://ceh.cedex.es/anuarioaforos/default.asp), *Agència Catalana de l’Aigua* (ACA; https://aca.gencat.cat); *Diputación Foral de Gipuzkoa* (DFG; https://www.gipuzkoa.eus/es/web/obrahidraulikoak); *Eaufrance (*https://www.hydro.eaufrance.fr*); Govern d’Andorra* (GA; https://www.govern.ad); *Banque nationale d’Accès aux Données sur les Eaux Souterraines* (ADES; https://ades.eaufrance.fr); *Confederación Hidrográfica del Ebro* (CHE; https://www.saihebro.com); and *Agencia Vasca del Agua / Uraren Euskal Agentzia* (URA; https://www.uragentzia.euskadi.eus/visor-de-estaciones-de-aforo/webura00-minima/es/). These organizations remain the owners of the original observations, which may be requested directly from them according to their respective data access policies. The published dataset does not redistribute the original observations but provides standardized hydrological indicators derived from them.

**Fig. 1:**
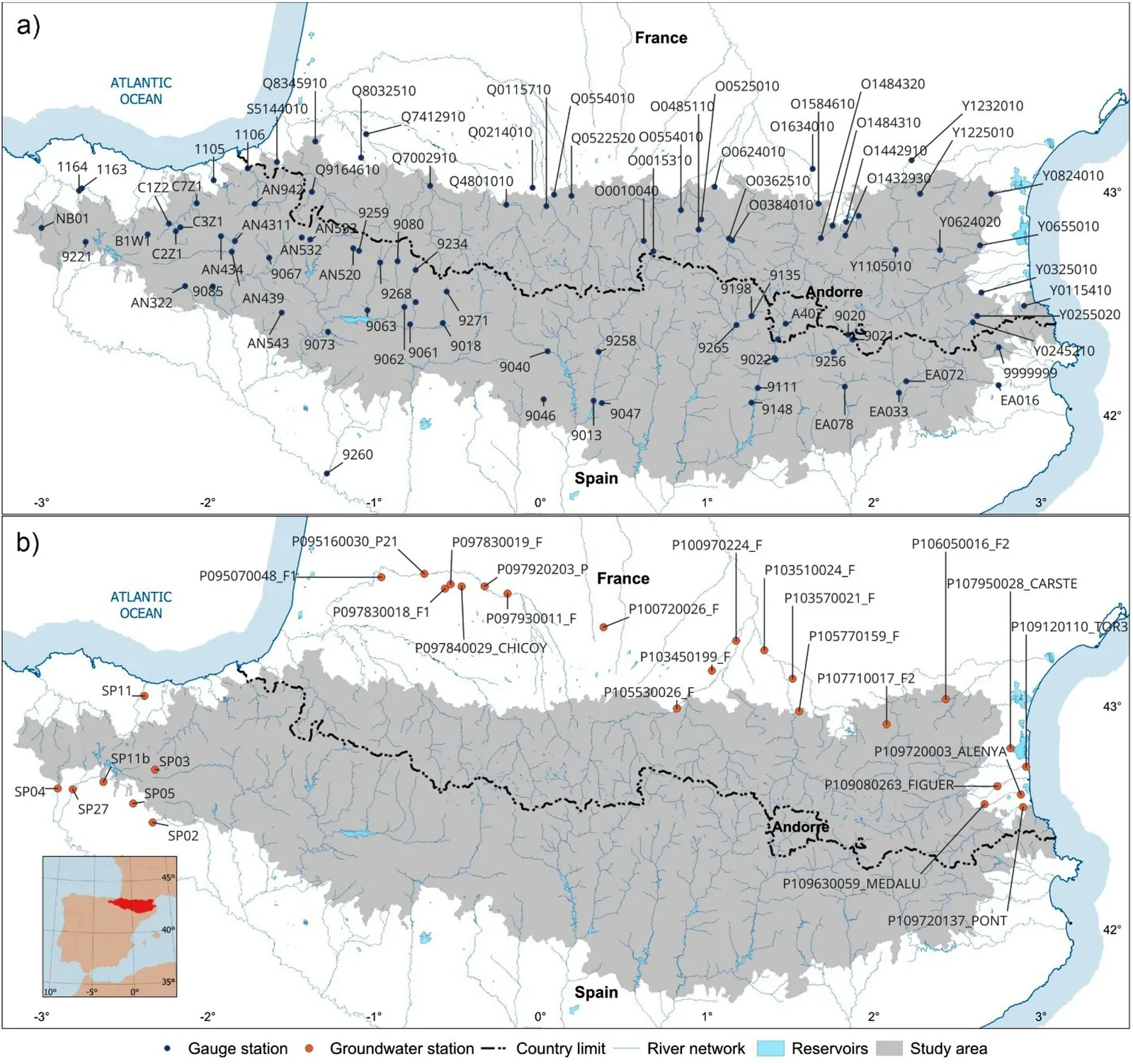
Location of the reference gauge sets in the study region: a) streamflow gauge stations; and b) groundwater stations with the location of the Pyrenees.

Streamflow data consists of regular daily resolution series, and the piezometric data contains irregular observations or monthly average values. Station selection was based on geographical, temporal and quality criteria. Only gauging stations located within the Pyrenean region, with records extending to the 2018–2019 hydrological year and containing at least 20 years of observations, were considered. Stations showing substantial anthropogenic influences, such as reservoir regulation, flow diversions or intensive water use, were excluded. A visual and statistical quality-control assessment was subsequently conducted to identify non-homogeneous records and anomalous behaviour. The resulting reference dataset comprised 93 streamflow gauging stations distributed across the Pyrenees. Thus, the analysis was performed on a reference set of gauging stations and another reference set of 29 piezometers (Fig. 1). Gauge catchment and aquifer boundaries were obtained from the PIRAGUA project GIS database.

A series of indicators describing flow regimes and water levels in aquifers were calculated from the raw data from the reference stations, using the piragua R package [7].

To characterize daily streamflow regimes and assess long-term changes across the Pyrenees, a set of non-parametric indicators was computed from daily discharge records. The median daily flow (*q50*) was selected as the main descriptor of average conditions, while percentiles 10 and 90 (*q10* and *q90*) represent low- and high-flow conditions, respectively. Additional percentiles (*q25* and *q75*) were calculated to provide intermediate flow thresholds.

Streamflow variability was quantified using the inter-decile range (*idr*), whereas the asymmetry and weight of the upper tail of the flow distribution were characterized by the inter-decile skewness (*dsk*) and inter-decile kurtosis (*dku*), respectively. These non-parametric statistics were preferred over parametric measures (e.g., mean or standard deviation) due to the characteristic non-normal distribution of streamflow data.

Two additional indices were included to describe extreme flow conditions. The baseflow index (*vcn3*) quantifies the intensity of low-flow periods, while the 20-year return level (*rl20*) represents high-flow extremes. The latter was estimated from annual and monthly series of observed maximum daily discharges using a non-stationary Generalized Extreme Value (GEV) model.

**Fig. 2:**
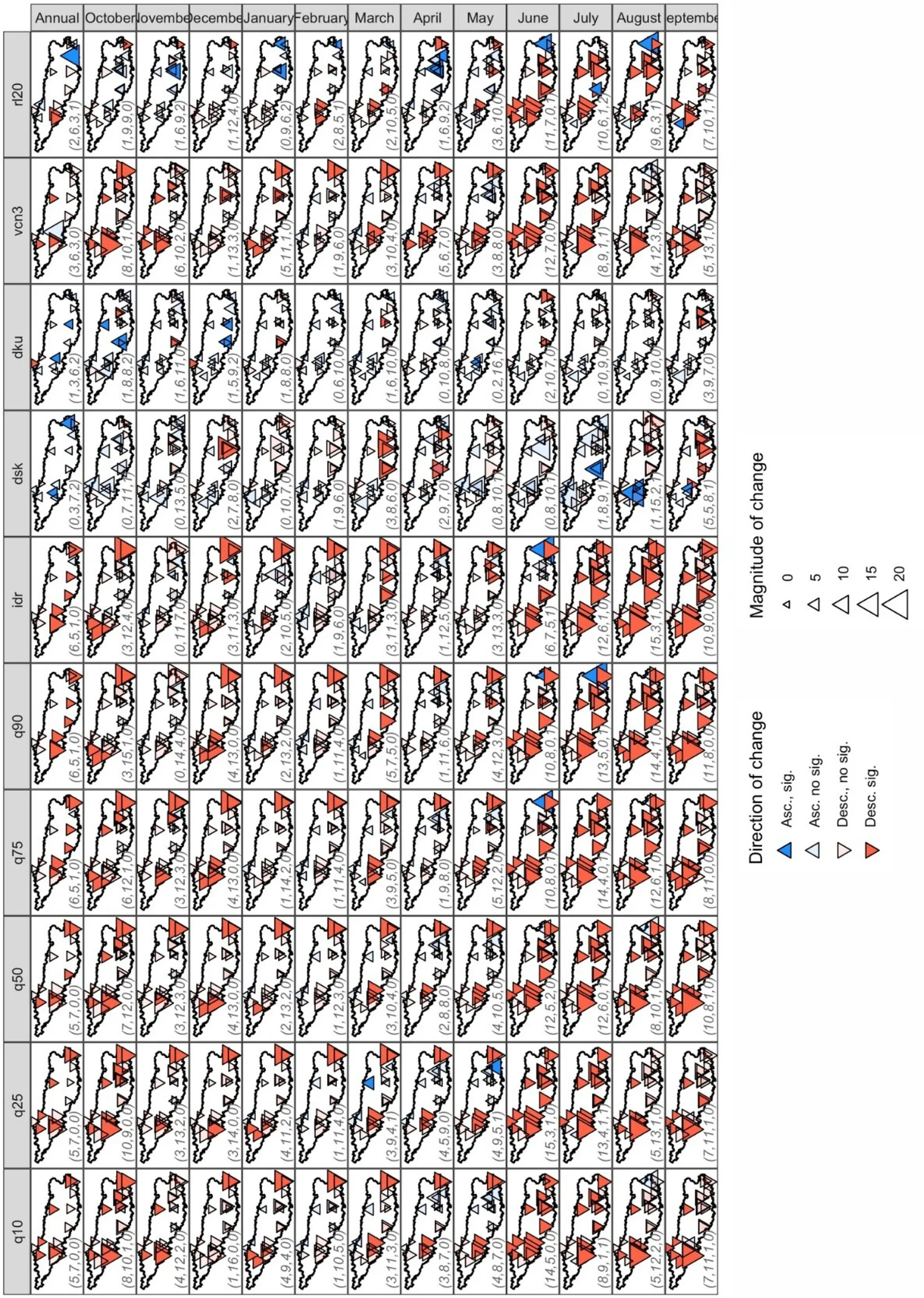
Map examples of flow trend indicators computed al annual and monthly scales for the period 1950-2019 with the Pyrenean area as spatial reference.

All flow indicators were computed at both annual and monthly scales (Fig. 2). Annual indicators correspond to hydrological years, defined as the period from 1 October to 30 September.

Unlike other studies, no data infilling was performed prior to the calculation of streamflow indicators. This decision was made to avoid the uncertainty and potential bias associated with reconstructing missing daily discharge values and to ensure that all computed indicators were based solely on observed data.

Because gaps exist in several of the observed time series—sometimes extending over multiple consecutive days—specific quality criteria were applied before computing annual and monthly statistics [1]. For annual indicators, years with more than 10 missing daily values or more than 5 consecutive missing days were automatically excluded. For monthly indicators, months with more than 5 missing (non-consecutive) values or more than 3 consecutive missing days were discarded.

Given the different lengths of the available time series, six temporal analysis windows ending in 2019 were defined: 1950-2019 (20 stations), 1960-2019 (26 stations), 1970-2019 (52 stations), 1980-2019 (58 stations), 1990-2019 (72 stations) and 2000-2019 (80 stations). Although shorter in length, the more recent periods include a larger number of gauging stations, allowing a comprehensive assessment of hydrological variability over the past seven decades using all available information.

To ensure data consistency, only time series meeting the following conditions were retained within each analysis period:

1. No more than one missing value per decade, after applying the above criteria for year or month removal.
2. No missing values at the beginning or end of the record, since these could significantly affect trend estimation.

For groundwater level records, the analysis focused on long-term trends in mean annual piezometric levels. This choice reflects the nature of the data, which represents groundwater table elevations, and their irregular temporal sampling, as measurements are often not taken at regular intervals. In addition, many observation wells present incomplete time series. Consequently, only annual mean levels were considered for trend analysis.

Mean annual groundwater levels were calculated for the period 1995–2019, using hydrological years defined as the period from 1 October to 30 September. Specific criteria were established to ensure data quality prior to analysis. The following records were excluded:

1. Years containing more than 100 missing measurements or more than 20 consecutive missing data.
2. Time series with more than four missing years within the 25-year analysis period (according to the above criteria).

Because the primary objective of the dataset was to assess temporal trends, series with missing data at the beginning or end of the record were also excluded from this analysis.

Trends in streamflow and groundwater levels indicators were computed to quantify long-term temporal changes across the study area. The slope of each trend was estimated using the non-parametric Theil–Sen estimator [8]. Statistical significance was evaluated with the Mann–Kendall test, after applying a pre-whitening procedure to account for temporal autocorrelation. The pre-whitening followed the Yue–Pilon method [9], which corrects for the inflation of significance levels that may occur in autocorrelated series and thus reduces the likelihood of false positives. Trend magnitudes were expressed as the relative change per decade, calculated as a percentage of the mean value for the analysed period.

Geographic layers were compiled and processed using QGIS (version 3.40.12).

## Limitations

Although the dataset provides a comprehensive overview of streamflow and groundwater level trends across the Pyrenees, several limitations should be considered. The spatial coverage of gauging stations and piezometers is heterogeneous (Fig. 1), and the temporal extent of records varies among sites. Some groundwater series present irregular or discontinuous measurements, which restricts the range of indicators that can be derived. No data infilling was performed, so the analyses rely exclusively on observed values, resulting in variable data availability among stations and periods. These limitations are inherent to long-term hydrological datasets but have been explicitly accounted for through strict quality control and selection criteria.

## Ethics Statement

We have read and followed the ethical requirements for publication in Data in Brief, and confirm that the current work does not involve human subjects, animal experiments, or any data collected from social media platforms.

## CRediT Author Statement

**Leticia Palazón:** Data curation, Visualization, Writing – review & editing; **Ane Zabaleta:** Data curation, Methodology, Formal analysis, Visualization, Writing – original draft; **Iñaki Antigüedad:** Conceptualization, Writing – review & editing; **Santiago Beguería:** Conceptualization, Methodology, Software, Formal analysis, Visualization, Writing – original draft, Funding acquisition; **Vivien Hakoun:** Data curation; **Pierre Le Cointe:** Data curation; **Luis Javier Lamban:** data curation; **Jorge Jódar:** data curation; **Yvan Caballero:** Conceptualization, Methodology, Formal analysis, Visualization Review & editing, Funding acquisition.

## Data Availability

DIGITAL.CSICPIRAGUA_indicators (Original data).
